# Effect of post-isometric relaxation versus myofascial release therapy on pain, functional disability, rom and qol in the management of non-specific neck pain: a randomized controlled trial

**DOI:** 10.1186/s12891-022-05516-1

**Published:** 2022-06-13

**Authors:** Zainab Khalid Khan, Syed Imran Ahmed, Aftab Ahmed Mirza Baig, Waqas Ahmed Farooqui

**Affiliations:** 1 Institute of Physical Medicine and Rehabilitation, Dow University of Health Sciences, Karachi, Pakistan; 2 Sindh Physical Medicine and Rehabilitation, Karachi, Pakistan; 3 School of Public Health, Dow University of Health Sciences, Karachi, Pakistan

**Keywords:** Non-Specific Neck Pain, Post Isometric Relaxation, Myofascial Release Therapy, Muscle Energy Technique, Isometric Strengthening Exercise

## Abstract

**Background:**

Non-specific neck pain is the most prevailing musculoskeletal disorder which has a large socioeconomic burden worldwide. It is associated with poor posture and neck strain which may lead to pain and restricted mobility. Physical therapists treat such patients through several means. Post isometric relaxation and Myofascial release therapy are used in clinical practice with little evidence to be firmed appropriately. So, this study was conducted to explore the effect of Post-isometric relaxation in comparison to Myofascial release therapy for patients having non-specific neck pain.

**Methodology:**

Sixty patients were randomly allocated to Post isometric group and the Myofascial group. The treatment period was of 2 weeks. All the patients were evaluated using the Visual analogue scale (VAS), Neck disability index (NDI), Universal Goniometer, and WHO BREF Quality of life-100 in the 1st and 6th sessions. Recorded data was entered on SPSS 21. Data were examined using two-way repeated ANOVA to measure the variance of analysis (group x time).

**Results:**

Analysis of the baseline characteristics revealed that both groups were homogenous in terms of age and gender i.e. a total of 60 participants were included in this research study 30 in each group. Out of 60 patients, there were 20(33.3%) males and 40(66.7%) females with a mean age of 32.4(5.0) years. Participants in the Post Isometric group demonstrated significant improvements (*p* < 0.025) in VAS, NDI, Cervical Extension, left side rotation ranges, and QoL (Social Domain) at the 2-week follow-up compared with those in the Myofascial group. In addition, the Myofascial group indicated significantly better improvement in the mean score of CROM (flexion and right and left side bending).

**Conclusion:**

The study demonstrated patients with nonspecific neck pain can benefit from the post isometric relaxation with significant improvement in pain, disability, cervical ROM, and Quality of life compared with myofascial release therapy.

**Trial registration:**

Clinical Trial registered on clinicaltrial.gov (NCT number) NCT04638062, 20/11/2020 (prospectively registered).

## Background

Non-specific neck pain (NSNP) is the most common and the 4^th^ leading cause of musculoskeletal disorder worldwide. It is estimated that about 70% of the population experiences neck pain throughout the life, with an annual incidence of 15% to 50%0 [1]. It is seen more commonly in middle-aged females [2]. It has been well established that NSNP is not only the risk factor for developing severe spinal pathologies and functional disability but that it is also associated with decreasing the quality of life and productivity of workers [3].

According to the global burden of diseases, the statistical prevalence of neck pain shows Asia at 10.14%, Australia at 10.13%, the Caribbean at 9.7%, Central Asia at 9.8%, Central Europe at 9.9%, East Asia at 11.8%, Eastern Europe as 9.9%, Latin America s 10.12%, and Southeast Asia as 7.6% [4]. According to the global burden of disease in the Mediterranean region the point prevalence of neck pain was estimated as 34.31 per 1000 in Pakistan [5].

The NSNP is described as pain present in the anatomical region of the neck without radiating to the upper limbs. It is also defined as the pain in the posterior region of the neck from the superior nuchal line to the spine of the scapula and the side region down to the superior border of the clavicle and the suprasternal notch [6]. The International Association for the Study of Pain refers to the duration of pain symptoms i.e. acute pain defined as pain for less than 7 days, sub-acute pain less than three months, and chronic pain more than three months [7]. NSNP is associated with occupational and musculoskeletal factors including prolonged work hours, poor sedentary lifestyle, high workload and demands, inappropriate computer workstation designs, and desk-bound work position [8, 9]. These factors may lead to muscle spasm, decreased cervical mobility and functional limitation [10].

Conservative treatment approaches to treat NSNP include pharmacological treatments such as analgesics and muscle relaxants [11, 12]. However, the physical therapy consists of manual therapy (post isometric relaxation, myofascial release), exercise therapy (stretching, strengthening, stabilization, endurance training), thermotherapy, cryotherapy [13], laser therapy, infra-red therapy, electrotherapy including TENS and ultrasound [14], dry needling [15], acupuncture [16]. The Post isometric relaxation is a form of muscle energy technique (MET) in which the patient’s muscles are moved in a particular direction against the counterforce of the therapist, which is mediated by Golgi tendon organ (GTO) when the muscle contracts isometrically. The GTO activates and responds by reflex inhibition and contracting antagonist muscles (by submaximal contraction of the muscles followed by stretching of the same muscles). It is used in the management of various musculoskeletal conditions that work on the principles of restoring biomechanics and reducing the movement restriction and pain [17]. The Post isometric relaxation involves the peripheral and central modulating mechanism by activating the muscles and joint mechanoreceptors, like periaqueductal grey in the midbrain or non-opioids noradrenergic descending inhibitory pathways and serotonergic. Further, MET increase augments hypoalgesia and fluid drainage. Rhythmic muscle contraction increases lymph flow rates and blood flow, while the mechanical forces acting on fibroblast increase transcapillary blood flow and produce changes in interstitial connective tissue. MET application may desensitize peripheral nociceptors and reduces the pro-inflammatory cytokines [18] Myofascial release therapy is the soft tissue technique that involves the application of low load and long duration stretch applied through knuckles or elbows on the restricted fascia that is facilitated by detecting the restriction in fascia [19] It decreases pain, increases blood flow and lymphatic drainage, and relaxes the muscles because the slow movement in the contracted muscles stimulates the parasympathetic nervous system that produces the feeling of relaxation [20].

According to a systemic review by Thomas et al. [21], studies reporting effectiveness of MET as standard treatment or combination with other therapeutic exercise is limited, the studies suggested that MET has a good clinical effect in reducing pain and improving ROM. However further research using a robust methodology is needed to enhance treatment effects for the management of NSNP. Overall, there is a lack of high-quality evidence investigating the effectiveness and safety of MET to guide its use in the clinical management of NSNP. However, the high risk of bias and methodological shortcomings require caution in interpreting these results [22]. Therefore, this study was conducted to evaluate the effect of post isometric relaxation versus myofascial release therapy on pain, functional disability, ROM, and QoL in the management of NSNP. In this study, the best manual technique is used with the good outcome effects in the physical therapy practices. This study not only helps the physical therapist to upgrade their knowledge regarding the NSNP but also provides the basis to manage the problem with more effective therapy.

## Methods

### Study design and participants

A single-blinded randomization was conducted at the Institute of Physical Medicine and Rehabilitation, Dow university of Health Sciences. In which, 60 participants with NSNP were recruited from November 2020 to November 2021. The following inclusion criteria were applied: Non-specific neck pain for 2–6 weeks, unilateral neck pain, patients' age group between 25 – 40, both genders male and female, and on visual analogue scale (VAS) pain intensity > 4 [8]. Participants were excluded if they presented with any serious pathology such as specific neck pain due to disc prolapsed, tumor of cervical spine, whiplash injury, cervical fractures, Cervicogenic headache, and any neurological signs consistent with nerve root compression [8].

### Sample size

The sample size for this study was calculated using Open EPI version 3. The effect size for the sample calculation was obtained from previous studies done on neck pain (8.03 ± 2.64) (11.27 ± 4.50) [23, 24]. Based on the data from these studies, the estimated sample of 21 per group was calculated with a 95% Confidence Interval and 80% Power of Analysis. To manage the dropout rate, the sample size was increased by 20%. Hence, the final sample size was 60 (30 in each group).

### Randomization and allocation

**Fig. 1:**
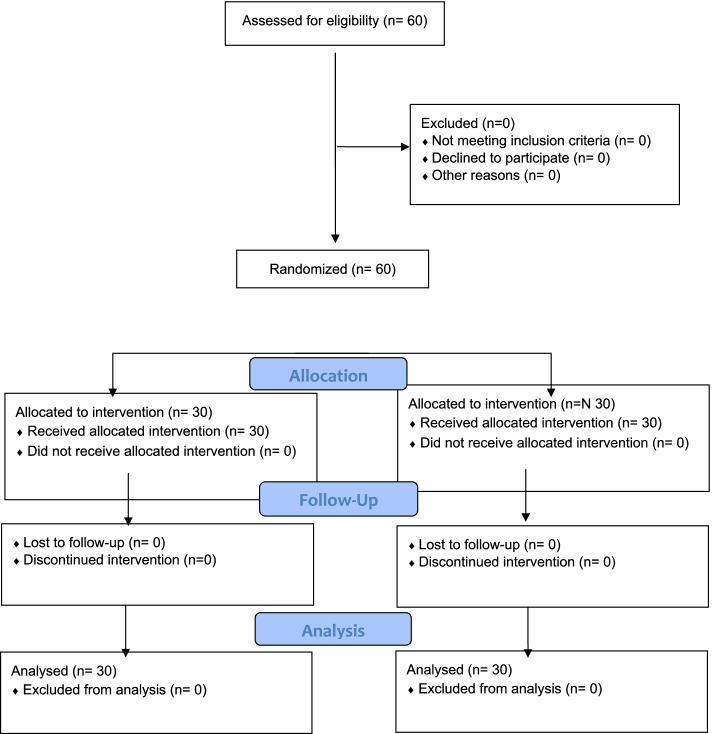
Flow chart of the recruitment, randomization and follow up of participant

The study subjects were randomly assigned into two groups A and group B (ratio 1:1) using a computer-generated random data sheet using www.random.org. The allocation was concealed using sequentially numbered sealed envelopes. This study was single-blinded as the participants were not aware of the treatment group to which they were assigned. The allocation was conducted by another researcher before the baseline, 60 participants were randomly assigned to either PIR (*n* = 30) and MFR (*n* = 30) groups (Fig. 1).

### Initial assessment

All participants underwent demographic information collection and physical examination. Patients who met the inclusion criteria were recruited, and assessment and screening were done based on physical examination and red flags for exclusion criteria by the physiatrist. The cervical spine was evaluated in sitting positions and passive physiological intervertebral movements such as flexion, extension, lateral flexion, rotation, and a passive accessory intervertebral motion test was conducted [25]. For neurological sign and symptoms upper limb tension test, spurling, distraction and compression test were performed [26].

### Outcome measure

#### Visual Analogue Scale (VAS)

It is a 10 cm or 100 mm psychometric response scale to measure pain intensity based on numerical values, anchored by a score of 0 no pain and score 10 worst ever pain. The VAS is a reliable and validated instrument for pain intensity with the interclass co- relation ranges between 0.95–0.98. The patients, who scored ≤ 3.4, were considered as mild pain, 3.5 to 7.4 as moderate pain, and ≥ 7.5 as severe pain [27].

#### Neck Disability Index (NDI)

NDI is a condition-specific, or patient completed a questionnaire comprising of 10 items to evaluate pain, and functional status which is mostly used for reporting neck pain. Each item on the scale is scored from 0–5, where all the scores are added to total points and interpreted as percentages i.e., 0 point or 0% means no activity limitation, and 50 points or 100% means complete activity limitation. The NDI is a reliable and valid questionnaire in neck pain patients with interclass co-relation ranges between 0.50–0.98. Patients scored between 0-4points (0–8%) were considered with no disability, patients scored between 5-14points (10 – 28%) were considered with mild disability, patients scored between 15-24points (30–48%) were considered with moderate disability, and patients scored between 25-34points (50- 64%) considered with severe disability, and patients scored between 35-50points (70–100%) were considered with complete disability [28].

#### Universal Goniometer (UG)

The universal goniometer (UG) is a measuring tool commonly used in clinics, to measure the active cervical range of motion (ACROM), UG was used to measure cervical ranges in all directions i.e., flexion, extension, lateral flexion, and rotation. To measure cervical flexion and extension the UG axis was placed over the external auditory meatus. The fixed arm was vertical and the movable arm was placed parallel on the imaginary line from the external auditory meatus to the base of nares. Assessor asked the patient to flex and extend the head and measured flexion and extension. For the lateral flexion, the UG axis was placed over the center of the sternal notch, the fixed arm was aligned parallel to the imaginary line passing between acromion processes, and the movable arm was aligned at the center of the nose. Assessor asked the patient to perform cervical lateral flexion side by side and measures. For the cervical rotation, the UG axis was placed over the center of the patient's head, fixed arm aligned parallel to an imaginary line passing between acromion process, and movable aligned at the tip of the nose. Assessor asked the patient to perform cervical rotation and measure. The intra-rater reliability for UG ranges from the intra-rater reliability for UG ranges from 0.80 to 0.98 and inter-rater reliability ranges from 0.83 to 0.98 [29].

#### WHO Quality of life-BREF (WHOQOL BREF)

The WHOQOL-BREF is an instrument that is used to assess psychometric properties. Comprising of 26 questions related to the following 4 domains: physical, psychological health, social relationship, and environment. Each item is rated on a 5-point Likert scoring scale where each score is transferred between 0 and 100. The interclass correlation coefficient shows 0.71–0.91. The scoring of WHOQOL BREF is performed for domains. The domain scoring is performed in a positive direction. The higher the facet scores the higher the QoL. The sum of the raw score is multiplied by 4 to make a score comparable to WHOQOL-100. Then the sum is converted into a 0–100 scale [30].

### Intervention

PIR group received post isometric relaxation for upper trapezius and levator scapulae along with cryotherapy and isometric strengthening exercises. MFR group also received myofascial release therapy for upper trapezius and levator scapulae muscles along with cryotherapy and isometric strengthening exercises. A total number of 6 interventional sessions was provided to each patient (3 sessions/weeks, for 2 weeks.) All the patients were re-assessed after the completion of 2 weeks.

#### Post Isometric Group (PIR)

##### Upper trapezius

Patient position and Therapist position: The patient was in a supine position, arm alongside the trunk, therapist was at the head side and stabilizes the shoulder using the palm of one hand, while the other hand was used to cup the ipsilateral mastoid process.

Line of movement: Neck flexion, contralateral full side bending followed by slight ipsilateral rotation was provided towards the side being treated. The patient was instructed to move the head back to the table and to shrug the stabilized shoulder with equal force against the therapist’s resistance to maintain the isometric contraction for 10 s. After the isometric contraction, the patient was asked to relax. Then, a gentle stretch was applied for 10 s with the same passive movement to reach a new muscle range.

**Fig. 2:**
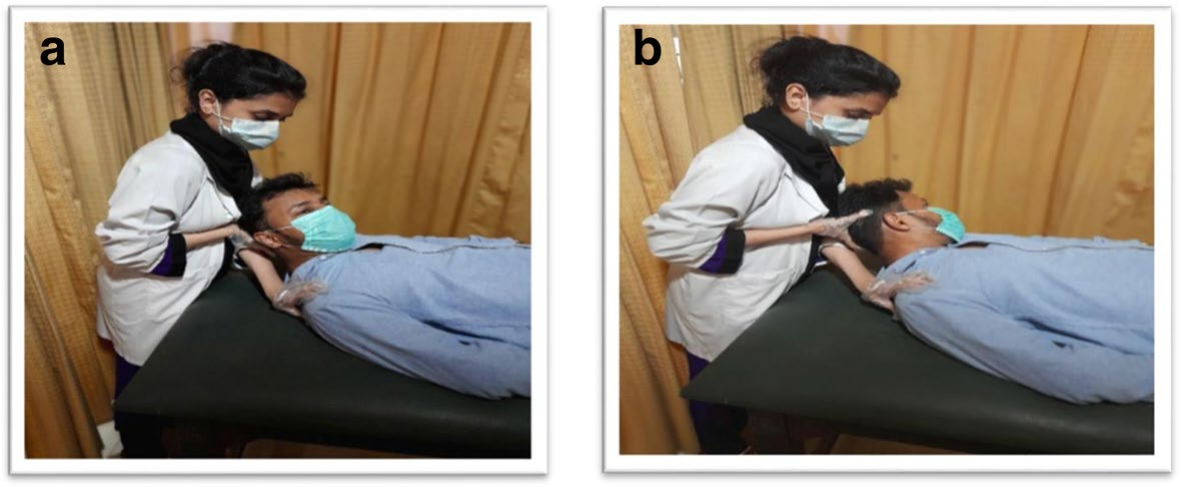
**A** PIR for Upper Trapezius (**B**) PIR for Levator scapulae

Repetitions: PIR on Trapezius was repeated 5 times [31]. (Fig. 2A).

##### Levator scapulae

Patient Position and Therapist Position: The patient was in a supine position with the arm been stretched out and supinated on the side to be treated, therapist was standing at the head side of the patient and place his opposite hand to stabilize the patient's shoulder so that therapist's forearm supports the patient’s neck, While the other hand supports the head.

Line of Movement: Neck into full flexion lateral flexion and rotation away from side to be treated was provided. The patient was instructed to move the head back to the table while at the same time slightly shrugging the shoulder with equal force against the therapist’s resistance to maintain the isometric contraction for 10 s. After the isometric contraction, the patient was asked to relax. Then, a gentle stretch was applied for 10 s with the same passive movement to reach a new muscle range.

Repetitions: PIR on Levator was repeated 5 times [31] (Fig. 2B).

#### Myofascial Release Therapy (MRF)

##### Upper trapezius

Patient Position and Therapist Position: The patient was in a sitting position, with hips being higher than the knee, feet slightly forward than knees, and well connected to the ground. The patient was informed to use feet and legs for back support. The therapist was standing at the backside of the patient.

Line of Movement: Myofascial release of the Trapezius was performed unilaterally with a soft fist, sinking and then taking up a line of tension from the mid-belly of the Trapezius, towards the acromion process, while the patient drops the head forward and slowly rotates the head from side to side. Then therapist provided resistance for 10 s to the contralateral side of rotation.

**Fig. 3:**
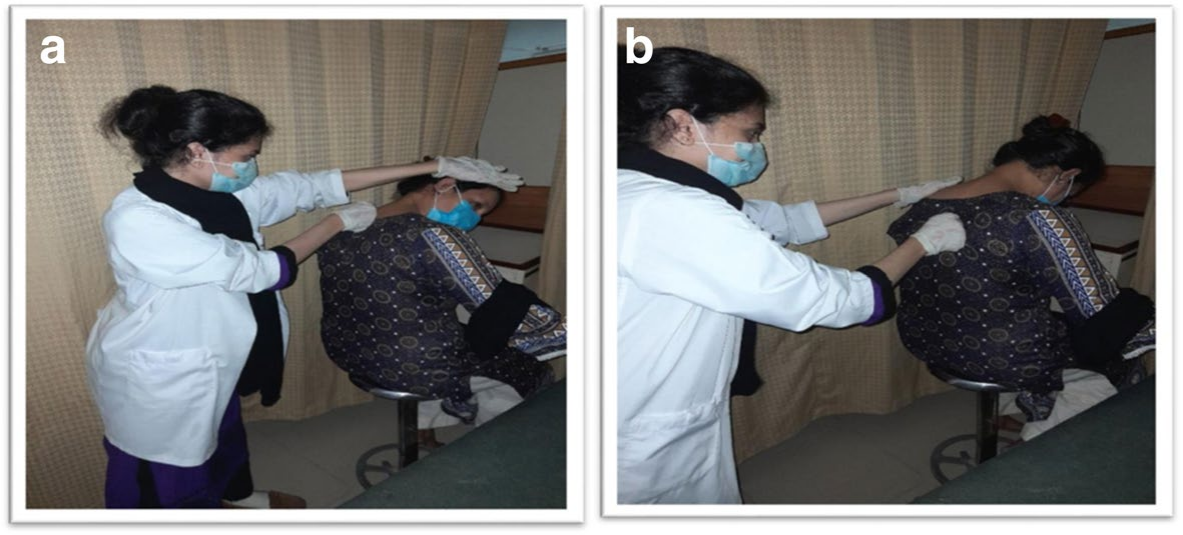
**A** MFR for Upper Trapezius (**B**) MFR for Levator scapulae

Repetitions: MFR on Trapezius was repeated 5 times [32]. (Fig. 3A).

##### Levator scapulae

Patient Position and Therapist Position: The patient was in a sitting position, with hips being higher to the knee, feet slightly forward than knees, and well connected to the ground. The patient was informed to use feet and legs for back support. The therapist was standing at the backside of the patient.

Line of Movement: The therapist applied the same unilaterally contract, but the line of tension was towards the inferior border of the scapula slightly lateral. Then therapist asked the patient to drop the head forward to increase the resistance for 10 s on Levator scapulae.

Repetitions: MFR on Levator was repeated 5 times [32]. (Fig. 3B).

### Conventional exercise

Both groups were also treated with conventional exercises, which included the isometric strengthening exercise which was performed on both groups in all six directions.

Procedure: Patient was in sitting position on a chair with feet flat on the ground, straight back and shoulder so that the weight is equal on both buttocks (Table 1).

**Table 1:** Isometric stengthning exercise [33]

MOVEMENT	METHOD	REPITATION
FLEXION	Patient was asked to press the palm against forehead, and to resist the forward movement for 10 s seconds	2 sets, 5 repetitions once a day
EXTENSION	Patient was asked to do the exercise again, pressing on the back of the head, and to resist the backward movement for 10 s seconds	2 sets, 5 repetitions once a day
LEFT SIDE BENDING	Patient was asked to do the exercise again, pressing on the left side of the head, and to resist the sideways movement for 10 s seconds	2 sets, 5 repetitions once a day
RIGHT SIDE BENDING	Patient was asked to do the exercise again, pressing on the right side of the head, and to resist the sideways movement for 10 s seconds	2 sets, 5 repetition once a day
LEFT ROTATION	Patient was asked to do the exercise again, pressing on the left side of the chin, and to resist the rotation movement for 10 s seconds	2 sets, 5 repetitions once a day
RIGHT ROTATION	Patient was asked to do the exercise again, pressing on the right side of the chin, and to resist the rotation movement for 10 s seconds	2 sets, 5 repetitions once a day

### Cryotherapy

The cryotherapy was applied for 10 min at the end of the treatment session.

### Statistical analysis

Data was analysed on Statistical Package of Social Sciences (IBM SPSS) Version 21. The descriptive statistics were performed initially which were characterized as age, gender, and duration of symptoms. For analysis of the distribution of age and duration of symptoms, the independent T-test was applied as per data distributed normally using the Shapiro Wilk test. However for the distribution of genders, the chi-square test was applied and for multiple comparisons of mean pain, functional disability, cervical ROM, and QoL scores within and between groups, the two-way repeated measure ANOVA was employed. A *p*-value < 0.025 was considered significant.

## Results

Analysis of the baseline characteristics revealed that both groups were homogenous in terms of age and gender, i.e. a total of 60 participants in this research study 30 in each group. Out of 60 patients, there were 20(33.3%) males and 40(66.7%) females with a mean age of 32.4(5.0) years. However, the baseline characteristic for both groups is presented in (Table 2).

**Table 2:** Baseline characteristics for the two groups

Baseline variable	Post isometric group (*n* = 30)	Myofascial group (*n* = 30)	*P*-value
Age (years)^a^	32.4 ± 4.7	32.4 ± 5.3	0.980^b^
Sex number (%)			
Male Female	10 (50) 20 (50)	10 (50) 20 (50)	0.068^c^
Duration of symptom (weeks)^a^	2.9 ± 0.9	3.1 ± 0.7	0.545^b^
VAS (0–10)	7.2 ± 1.2	7.0 ± 1.2	0.459^b^
NDI (0–50)	20.6 ± 4.9	21.6 ± 6.0	0.728^b^
Flexion(^0^)	33.8 ± 5.7	33.1 ± 5.7	0.623^b^
Extension(^0^)	34.8 ± 5.1	32.6 ± 6.7	0.131^b^
Right side bending(^0^)	28.8 ± 6.9	26.8 ± 7.3	0.282^b^
Left side bending(^0^)	29.2 ± 6.7	28.5 ± 8.1	0.706^b^
Right rotation(^0^)	63.0 ± 9.3	63.4 ± 9.3	0.847^b^
Left rotation(^0^)	66.5 ± 8.8	64.9 ± 8.7	0.483^b^

*VAS* Visual analogue scale, *NDI* Neck disability index

^a^Values are mean and standard deviation

^b^Independent t test

^c^Chi-square was used for association

In this study, the pain intensity on the visual analogue scale showed a significant group-by-time interaction (F = 6.113, *p* = 0.008, ηp2 = 0.095) with the PIR group indicating better pain reduction with mean VAS (1.3 ± 1.0) over time than those in MFR group (2.0 ± 1.2). The neck disability index also showed a significant group by time interaction (F = 8.844, *p* = 0.002, ηp2 = 0.132) with the PIR group indicating better improvement in mean NDI (1.4 ± 2.0) over time than those in MFR group (3.6 ± 3.4). (Table 3).

**Table 3:** Outcome data for pain intensity and disability

**Outcome Measures**	**Baseline**^**a**^	**2 weeks**^**a**^	**Mean within group differences**^**b**^ **Baseline to 2 weeks**	**Mean between group differences **^**b**^ **Baseline to 2 weeks**
**VAS(0–10)**				
PIR group	7.2 ± 1.2	1.3 ± 1.0	5.9(*p* < 0.001)	
				-0.7(*p* = 0.008)
MRF group	7.0 ± 1.2	2.0 ± 1.2	5.0(*p* < 0.001)	
**NDI (0–50)**				
PIR group	20.6 ± 4.9	1.4 ± 2.0	18.0(*p* < 0.001)	
				-2.2(*p* = 0.002)
MFR group	21.6 ± 6.0	3.6 ± 3.4	17.5(*p* < 0.001)	

PIR Post isometric relaxation, MFR Myofascial release, VAS Visual analogue scale, NDI Neck disability index

^a^Values of mean and standard deviation

^b^Values of mean difference and ***p***-value

CROM shows statistically significant change within mean scores of both the PIR group (Extension, Right and Left rotation) and MFR group (Flexion, Right and Left side bending), while pairwise comparison between PIR and MFR groups shows statistical significance in cervical extension and left rotation. The active cervical extension showed a significant group-by-time interaction with (F = 5.716, *p* = 0.010, ηp2 = 0.090) with the PIR group indicating significantly better improvement in mean cervical extension ROM (43.4 ± 1.6) over time than those in MFR group (42.0 ± 2.7). Furthermore, the left rotation also showed a significant group-by-time interaction with (F = 5.378, *p* = 0.012, ηp2 = 0.085) with the PIR group indicating significantly better improvement in mean cervical rotation ROM (79.2 ± 2.2) over time than those in MFR group (76.5 ± 5.9). However, no significant group-by-time interaction was observed for CROM, as measured using cervical flexion, right side bending, left side bending, and right rotation. (Table 4).

**Table 4:** Within group change score and pairwise comparison of between group differences for cervical range of motion

**Outcome** **measures**	**Baseline**^**a**^	**2 weeks**^**a**^	**Mean within group differences**^**b**^ **Baseline to 2 weeks**	**Mean between group differences**^**b**^ **Baseline to 2 weeks**
**Flexion (**^**0**^**)**				
PIR group	33.8 ± 5.7	44.6 ± 1.3	-10.8(*p* < 0.001)	
				-0.6(*p* = 0.842)
MRF group	33.1 ± 5.7	44.6 ± 1.2	-11.5(*p* < 0.001)	
**Extension (**^**0**^**)**				
PIR group	34.8 ± 5.1	43.4 ± 1.6	-8.6(*p* < 0.001)	
				1.4(*p* = 0.010)
MRF group	32.6 ± 6.7	42.0 ± 2.7	-9.4(*p* < 0.001)	
**Right Side Bending (**^**0**^**)**				
PIR group	28.8 ± 6.9	43.7 ± 2.1	-14.9(*p* < 0.001)	
				0.9(*p* = 0.937)
MRF group	26.8 ± 7.3	42.7 ± 2.4	-15.9(*p* < 0.001)	
**Left Side Bending (**^**0**^**)**				
PIR group	29.2 ± 6.7	43.1 ± 2.4	-14.5(*p* < 0.001)	
				-1.1(*p* = 0.982)
MRF group	28.5 ± 8.1	44.3 ± 1.7	-15.8(*p* < 0.001)	
**Right Rotation (**^**0**^**)**				
PIR group	63.0 ± 9.3	79.1 ± 2.2	-16.1(*p* < 0.001)	
				1.4(*p* = 0.062)
MRF group	63.4 ± 9.3	77.6 ± 4.6	-14.2(*p* < 0.001)	
**Left Rotation (**^**0**^**)**				
PIR group	66.5 ± 8.8	79.2 ± 2.2	-12.7(*p* < 0.001)	
				2.7(*p* = 0.012)
MRF group	64.9 ± 8.7	76.5 ± 5.9	-11.6(*p* < 0.001)	

*PIR* Post isometric relaxation, *MFR* Myofascial release, *CROM* Cervical range of motion

(.^0^)Ranges in degree

^a^Values of mean and standard deviation

^b^Values of mean difference and *p*-value

WHOQOL-BREF-100 shows statistically significant change within all domains of QoL in both PIR and MFR groups, while pairwise comparison between PIR and MFR groups in the social domain of WHO quality of life brief-100 shows statistical significant group by time interaction (F = 4.796, *p* = 0.016, ηp2 = 0.076) with the PIR group indicating significantly better improvement with a mean score of the social domain (55.6 ± 18.0) over time than those in MFR group (64.5 ± 13.1)). However, no significant group-by-time interaction between groups was observed in the physical, psychological, and social domains. (Table 5).

**Table 5:** Within group change score and pairwise comparison of between group differences for quality of life

**Outcome** **measure**	**Baseline**^**a**^	**2 weeks**^**a**^	**Mean within group differences**^**b**^ **Baseline to 2 weeks**	**Mean between group differences**^**b**^ **Baseline to 2 weeks**
**Physical Domain**				
PIR group	41.2 ± 8.9	84.4 ± 8.8	-43.2(*p* < 0.001)	
				-0.1(*p* = 0.472)
MFR group	43.4 ± 10.8	84.5 ± 9.3	-41.1(*p* < 0.001)	
**Psychological Domain**				
PIR group	40.0 ± 14.1	83.9 ± 6.2	-43.9(*p* < 0.001)	
				-0.1(*p* = 0.472)
MFR group	45.1 ± 16.9	84.5 ± 9.1	-39.4(*p* < 0.001)	
**Social Domain**				
PIR group	40.2 ± 19.2	55.6 ± 18.0	-15.4(*p* < 0.001)	
MFR group	54.3 ± 17.0	64.5 ± 13.1	-10.2(*p* < 0.001)	-8.9(*p* = 0.016)
**Environmental Domain**				
PIR group	56.4 ± 12.5	76.1 ± 7.9	-19.7(*p* < 0.001)	
				-4.6(*p* = 0.980)
MFR group	58.1 ± 12.1	80.7 ± 8.9	-22.5(*p* < 0.001)	

WHOQOL-BREF World health organization quality of life questionnaire

^a^Values of mean and standard deviation

^b^Values of mean difference and *p*-value

## Discussion

The current single-blinded RCT was designed to evaluate the effect of post isometric relaxation vs. myofascial release therapy on pain, functional disability, ROM, and QoL in the management of non-specific neck pain. The results of this study revealed that PIR showed significant improvements in pain, disability, Cervical ROM, and QoL at the 2-week follow-up compared with those in the Myofascial group.

In the current study, within and between groups analyses of VAS showed significant relief of pain after 6 sessions of treatment application in both groups. However, the PIR group was shown to be superior in means scores to the MFR group statistically. The clinically significant result could be interpreted because the greater change in VAS scores within the PIR group than MFR is within the range of a minimal clinical important difference (MCID) of VAS-neck ranging from 4.6 to 21.4 [34]. The reduction of pain following application of PIR could be due to the inhibitory effects of Golgi tendon organs, which reduces the motor neuronal discharges, thereby causing relaxation of the musculotendinous unit by resetting its resting length and Pacinian corpuscle modification. These reflexes allow relaxation in musculotendinous unit tension and decreased pain perception [35]. The results of our study were in agreement with the study conducted to evaluate the effect of PIR on 30 patients diagnosed with mechanical NP showed that PIR has a significant effect in pain reduction and increasing ROM [36]. The current study was also in consistence with the finding of the study in which MET was compared with manual pressure release to treat mechanical neck pain on 45 females participants. The finding of the study shows statistically significant improvement in within group analysis of pain intensity, PPT, CROM and disability *p* < *0.05* in both groups. However, between group showed insignificant results *p* > *0.05* in MPR group, whereas MET shows marginal significance in only in pain intensity *p* < *0.05 *[24].

Regarding the comparison between the experimental treatments, we found that disability, measured through NDI, showed statistically significant differences in the immediate effects in favour of the PIR group. In the short term, there were also statistically significant differences between the experimental groups, this suggests that PIR is faster in obtaining a decrease in cervical disability. According to a systematic review, it was concluded that NDI has an MDC of 10% and MCID of 14% [37]. According to the present study, the average change score of both groups exceeded MDC and MCID values (i.e. mean difference within the groups on NDI was PIR 18.7 and MFR 17.5). Therefore, the difference in improvement between groups was significant, the clinical importance was also certain when the interpretation was performed based on mean difference. this could be because the NDI assesses different aspects of neck pain which consists of pain intensity, and daily activities, suggesting that improvement in the score might be due to the reduction of pain. The results of this study were in line with the study conducted by Phadke et al. conducted an RCT on 60 individuals with NSNP, and indicated that 6 days of manual therapy including MET and static stretching along with strengthening exercise and hot pack causes a significant improvement on both groups. However, both VAS and NDI scores showed better improvement in the MET group as compared to the stretching group (*p* < 0.025) [23].

The comparison of CROM within both groups showed significant improvement in all six movements. However pairwise comparison between groups also showed equal improvement in both groups. For patients with NP, MCID value had a variation of 5 to 10^0 ^[38]. In the present study the mean difference score in all CROM has reached that MCID value giving clinically significant result. PIR showed better improvement in (Extension, Right and Left rotation), this could be explained by the hypothesis suggested by Taylor *et al.* that a combination of contractions and stretches (as used in PIR) might be more effective in producing viscoelastic changes than passive stretching alone, because the greater forces produce increased viscoelastic change and passive extensibility [39]. However, the MFR group also showed improvement in (Flexion, Right and Left side bending), studies showed the increase in the ROM from MFR occur by improving joint hypo mobility and breaking the adhesion between the soft tissue by shearing the crosslink’s and remobilizing the fascia back into the gel-like structure [32] The results of the present study were in agreement with the study conducted by Gilani et al. They randomly divided 30 patients into two groups: Group 1 (*n* = 15) received ischemic compression (IC) in upper trapezius myofascial trigger points, and Group 2 (*n* = 15) received MET. According to this study, IC was more significantly effective (*p* ≤ 0.001) for reducing neck pain than MET. MET, in contrast, was more effective for improving range of motion (*p* ≤ 0.001) [40]. Another RCT conducted by huguet to evaluate the effect of myofascial release therapy on neck pain including 41 participants diagnosed with NSNP. The result of this stated that myofascial release could be a better than multimodal physical therapy program in improving pain and CROM [41]

Within the available literature and for our knowledge this is the first study which investigate the effect of PIR as compared to MFR by using WHO-BRIEF 100 scale to access the quality of life in NSNP patients. According to authors’ knowledge, no consensus is available regarding the MCID values of WHO BREF-100 in NP yet. However, a study on NSNP reported MCID as 1.80 for Physical domain, 1.68 for physiological domain, 2.48 for social relationship, and 1.68 environmental domain [42]. In this study, among the four domains of WHO-BRIEF 100 all domains had a clinical change of scores within groups with MCID. Only the social domain showed a statistically significant difference between groups with more clinical differences. Whereas, the study conducted by Rodrigues et al. on 59 patients observed that both therapeutic procedures manual therapy and myofascial release improves the QoL of patients with occupational mechanical NP by using SF 36. The manual therapy group showed significant changes only in the dimensions of physical functioning and bodily pain, whereas the myofascial group achieved significant improvements in both PCS and MCS (physical and mental component scores). This study's results were in contrast with the finding of the above study, as in our study PIR was shown to be statistically superior compared to MFR [43].

In addition, the study limitation is the absence of a long-term evaluation of the outcome. Other studies with larger follow-up periods along with control groups are needed to determine the long-term clinical benefit and to generalize the intervention results. The main strength of the study is the clear and understandable methodology for both of the treatment groups and secondly, this study was single-blinded RCT.

### Clinical implication

NSNP is an integral health problem that plays a major role in developing cervical pathology and results in an enormous burden of disease in society. Simple and safe treatment procedures like PIR and MFR combined with other conventional therapies like isometric strengthening exercises and cryotherapy could be a great way out. It provides an inexpensive and easy way of treatment in patients with NSNP. Our study results suggest that a combination of isometric strengthening exercise and cryotherapy with PIR and MFR can potentially reduce NSNP and improves the QoL.

## Conclusion

The study demonstrated both the manual therapy techniques were effective in alleviating NSNP. However, individuals with non-specific neck pain who received PIR showed an overall better outcome in terms of VAS, (cervical extension and rotation), NDI, and WHO QoL BREF 100 (Social domain) than those who received the MFR at 2 weeks.

## Data Availability

Data included in the current study are not publicly available to ensure confidentiality of the patients but are available from the corresponding author on reasonable request.

## Abbreviations

ACROM: Active Cervical Range of Motion
ANOVA: Analysis of Variance
CROM: Cervical Range of Motion
DALY: Disability Adjusted life years
DUHS: Dow University of Health Sciences
GTO: Golgi Tendon Organ
HRQoL: Health Related Quality of Life
IPM&R: Institute of Physical Medicine and Rehabilitation
IRB: Institutional Review Board
MET: Muscle Energy Technique
MFR: Myofascial Release Therapy
NDI: Neck Disability Index
NSNP: Non-Specific Neck Pain
PIR: Post-Isometric Relaxation
QoL: Quality of Life
RCT: Randomized Controlled Trial
ROM: Range of Motion
UG: Universal Goniometer
VAS: Visual Analogue Scale
WHOQOL BREF 100: World Health Organization Quality of Life BREF 100
YLD: Year Lived with Disability
